# An assessment tool for computer-assisted semen analysis (CASA) algorithms

**DOI:** 10.1038/s41598-022-20943-9

**Published:** 2022-10-07

**Authors:** Ji-won Choi, Ludvik Alkhoury, Leonardo F. Urbano, Puneet Masson, Matthew VerMilyea, Moshe Kam

**Affiliations:** 1 Department of Electrical and Computer Engineering, New Jersey Institute of Technology, Newark, NJ 07102 USA; 2 Department of Electrical and Computer Engineering, Drexel University, Philadelphia, PA 19104 USA; 3 Penn Fertility Care, Hospital of the University of Pennsylvania, Philadelphia, PA 19104 USA; 4 Ovation Fertility, Brentwood, TN 37027 USA

**Keywords:** Biotechnology, Computational biology and bioinformatics, Health care, Medical research

## Abstract

Computer-Assisted Semen Analysis (CASA) enables reliable analysis of semen images, and is designed to process large number of images with high consistency, accuracy, and repeatability. Design and testing of CASA algorithms can be accelerated greatly if reliable simulations of semen images under a variety of conditions and sample quality modes are available. Using life-like simulation of semen images can quantify the performance of existing and proposed CASA algorithms, since the parameters of the simulated image are known and controllable. We present simulation models for sperm cell image and swimming modes observed in real 2D (top-down) images of sperm cells in laboratory specimen. The models simulate human sperm using four (4) types of swimming, namely linear mean, circular, hyperactive, and immotile (or dead). The simulation models are used in studying algorithms for segmentation, localization, and tracking of sperm cells. Several segmentation and localization algorithms were tested under varying levels of noise, and then compared using precision, recall, and the optimal subpattern assignment (OSPA) metric. Images of real human semen sample were used to validate the segmentation and localization observations obtained from simulations. An example is given of sperm cell tracking on simulated semen images of cells using the different tracking algorithms (nearest neighbor (NN), global nearest neighbor (GNN), probabilistic data association filter (PDAF), and joint probabilistic data association filter (JPDAF)). Tracking performance was evaluated through multi-object tracking precision (MOTP) and multi-object tracking accuracy (MOTA). Simulation models enable objective assessments of semen image processing algorithms. We demonstrate the use of a new simulation tool to assess and compare segmentation, localization, and tracking methods. The simulation software allows testing along a large spectrum of parameter values that control the appearance and behavior of simulated semen images. Users can generate scenarios of different characteristics and assess the effectiveness of different CASA algorithms in these environments. The simulation was used to assess and compare algorithms for segmentation and tracking of sperm cells in semen images.

## Introduction

Computer-Assisted Semen Analysis (CASA) systems and their algorithms continue to be of great interest to clinicians and andrology researchers^1^. Modern CASA systems “have been designed to objectively and quantitatively measure several aspects of sperm structure and function, aiming to provide high levels of intra- and inter-laboratory consistency”^1,2^. To achieve this aim, methods of noise filtering, image segmentation, localization, multi-object tracking, and machine learning were employed^3–15^.

A major challenge in developing and validating CASA systems is the accurate assessment and comparison of their semen analysis methods to the ground truth. In order to validate a CASA system, this process needs to take place across a representative sample of the expected semen images. For real-life samples, the ground truth is often unknown, motivating the use of high-quality image simulations with modifiable parameters for validation of CASA systems and algorithms. Simulations of this kind have the potential to help in developing automated semen analysis systems, and in comparing different candidate algorithms to each other.

To generate a simulation of semen sample video for assessment and validation, we need the following: *(1) a model for the image of a sperm cell* (to generate sperm cells in the simulated semen image) and *(2) a model for the sperm cell movement* that defines how the semen image changes over time. The development of sperm cell model in this paper mirrors similar work that was done for other cell types^16–21^. The development of sperm cell swim models is informed by several existing approaches. Some of these use a set of nonlinear equations of motion (Armon et al.^22^ and Ch. 6 of Urbano (2015)^4^). Others use fluid dynamics to develop a more refined description of cell movements^23–31^. However, most existing simulation models of cell dynamics^25–31^ did not integrate multiple cells into a comprehensive image such as the ones used in practice by CASA systems^32,33^. Integration of these single cell descriptions into full-scale multi-cell images is required before relevant image simulations can be developed and used.

**Figure 1:**
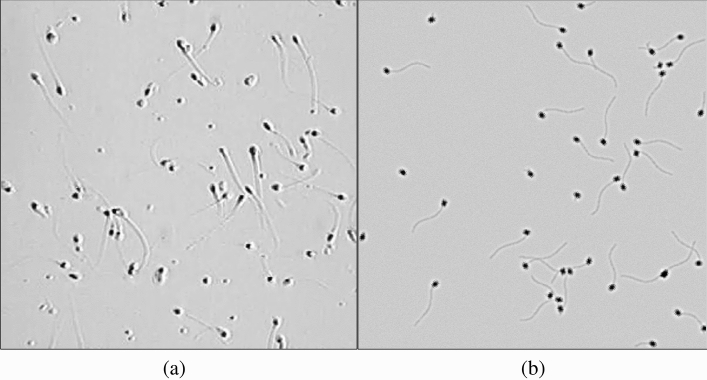
(**a**) Image of real human semen sample. (**b**) Image of simulated semen sample.

In this paper, we provide models for the image of a sperm cell and for four (4) different swimming modes of sperm cells (circular, linear mean, hyperactive, and immotile). The computational requirements to simulate these models and integrate them into multiple-cell image are relatively low, allowing for generation of multiple images in a format similar to the images used by CASA systems. As an example, a snapshot of a real semen image and simulated image are shown side by side in Fig. 1. Figure 1a is an image of semen sample at $200 \times$ magnification. Figure 1b is a simulated semen image generated by using parameters estimated from the semen image on the left.

The rest of this paper is organized as follows. In the “Methods” section, we present a model of a sperm cell and of the swimming modes. In the “Testing setup” section, we explain how simulated images were created to mimic real ones for visual and numerical comparisons. In the “Testing” section, we demonstrate use-cases for segmentation, localization, and tracking algorithms.

This paper is accompanied by simulation software which is publicly available at Choi et al.^34^ as a stand-alone software and as MATLAB codes (https://github.com/JiwonChoi-NJIT/NJIT_sperm_simulator). The dataset supporting the conclusions of this article are available in the github repository (https://github.com/moshekam/NJIT-Semen-Images-Data-Fusion-Lab)^35^.

## Methods

**Figure 2:**
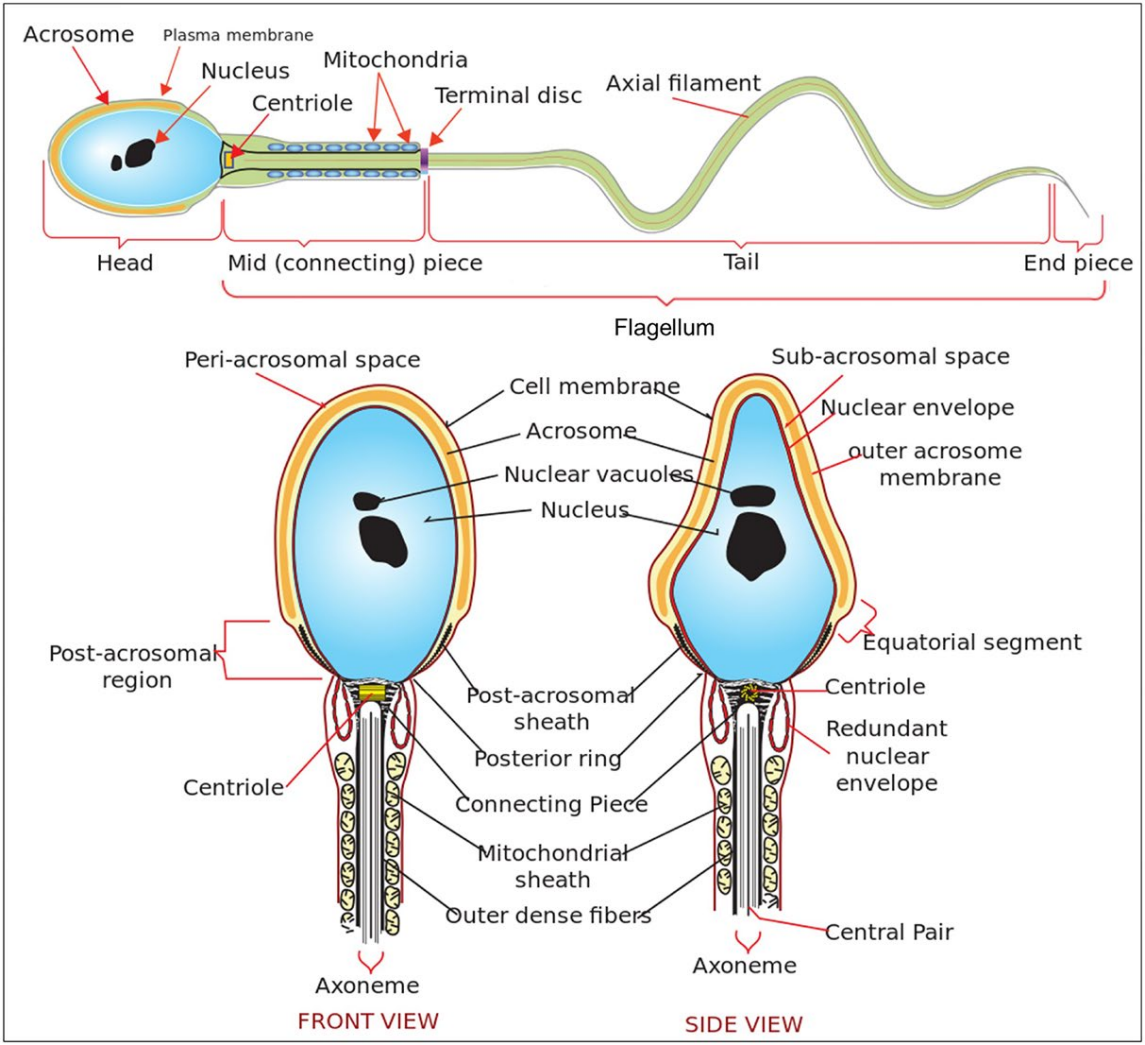
Diagram of a human sperm cell^36^. (Top) Main components of a sperm cell. (Bottom) Front and side view of sperm head and midpiece.

The human sperm cell (see Fig. 2, retrieved from Villarreal (2006)^36^) has two main parts, the head and the flagellum. The flagellum is composed of midpiece, tail (also called principal piece), and end piece. The flagellum moves in a wave-like motion to propel the cell^37^. In the following sections we describe the 2-dimensional (2-D) image of the head and the flagellum of a sperm cell, and the models of sperm movements.

### Simulating a sperm cell

We generate the image of the head and the flagellum of a sperm cell separately, and then combine them. In modeling the head and the flagellum of a sperm cell, we follow the morphology of sperm cells described in the WHO laboratory manual for the examination and processing of human semen^38^. The normal shape of the human sperm head is generally oval. The tail, or the principal piece, of the flagellum is a thin cylinder of uniform calibre. In our simulation, the flagellum is modeled by the tail only (we do not consider the midpiece or the end piece for the simulation model since they are often too small to be observed separately in the resolution used by CASA systems).

**Figure 3:**
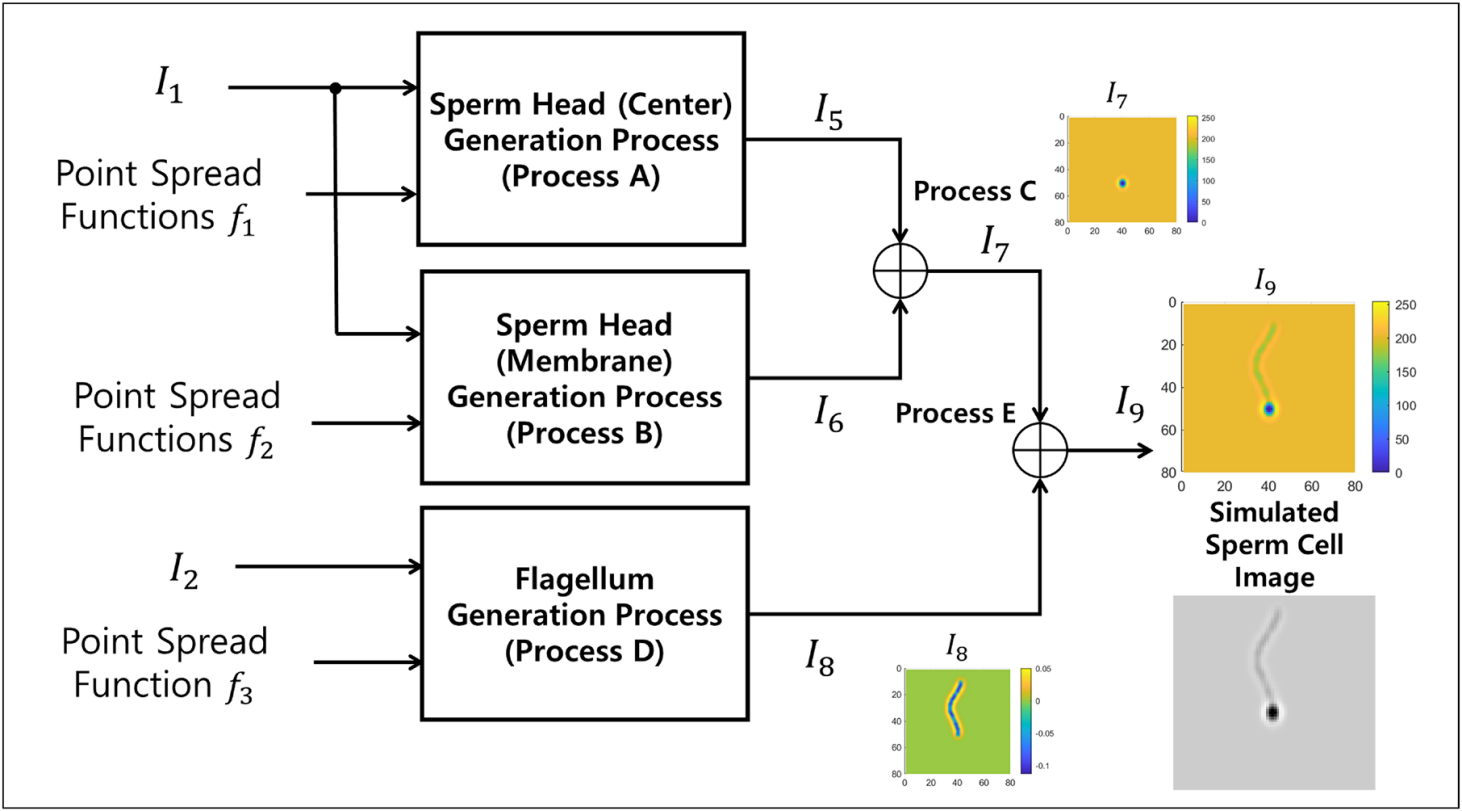
Flowchart of sperm image generation process. Image $I_1$ and $I_2$, and point spread functions $f_1$, $f_2$, and $f_3$ are the inputs. The output $I_9$ is the simulated sperm image. Details of each process are shown in Figs. 5, 7, 8, 9, and 10. Image $I_7$ shows the image of sperm head (Output of process C). Image $I_8$ shows the image of flagellum (Output of process D). Image $I_9$ shows the image of sperm head (Output of process E). Color bars next to $I_7$-$I_9$ indicate the corresponding grayscale intensity values. Resulting grayscale image of simulated sperm cell below the colored plot of $I_9$.

The flowchart of the process of generating the sperm image is shown in Fig. 3. The inputs of the process are images $I_1$ (corresponding to the sperm head) and $I_2$ (corresponding to the curve of the flagellum), and point spread functions $f_1$, $f_2$, and $f_3$. The image of sperm head center $I_5$ and membrane $I_6$ are generated using the operations labeled Process A and B, respectively. The inputs for Process A are image $I_1$ and point spread function $f_1$, and the inputs for Process B are image $I_1$ and point spread function $f_2$. The images of sperm head center $I_5$ and membrane $I_6$ are merged in Process C, producing a final image of sperm head $I_7$. Processes A-C are therefore the sperm head generation process. The final image of the flagellum $I_8$ is generated using Process D, whose inputs are image $I_2$ and point spread function $f_3$. Lastly, the image of sperm head $I_7$ and flagellum $I_8$ are merged in Process E, producing the complete image of the sperm cell, $I_9$. A grayscale image of the simulated sperm cell is shown just below the full-color simulated cell image in Fig. 3.

$I_1$ is an image of *N* by *N* pixels where

1 $$\begin{aligned} I_1(x,y,t) = {\left\{ \begin{array}{ll} 255 &{} (x,y)\in \{(x_{H_C}(t),y_{H_C}(t)), (x_{H_L}(t),y_{H_L}(t)),\\ &{} \qquad \qquad (x_{H_H}(t),y_{H_H}(t)), (x_{H_I}(t),y_{H_I}(t))\} \\ 0 &{} \text {otherwise}. \end{array}\right. } \end{aligned}$$

*N* is the size of the frame of the simulation image. Here, $\{(x_{H_C},y_{H_C})$, $(x_{H_L},y_{H_L})$, $(x_{H_H},y_{H_H})$, $(x_{H_I},y_{H_I})\}$ are the locations of sperm heads of cells engaged in circular swimming, linear mean swimming, hyperactive swimming, or no swimming (immotile cells), respectively. $I_2$ is also an image of *N* by *N* pixels where

2 $$\begin{aligned} I_2(x,y,t) = {\left\{ \begin{array}{ll} 255 &{} (x,y)\in \{(x_{tail_C}(k,t),y_{tail_C}(k,t)), \; (x_{tail_L}(k,t),y_{tail_L}(k,t)), \\ &{} \qquad \qquad (x_{tail_H}(k,t),y_{tail_H}(k,t)), \; (x_{tail_I}(k,t),y_{tail_I}(k,t))\}, \qquad k = 1,2,3,\ldots ,M, \\ 0 &{} \text {otherwise.} \end{array}\right. } \end{aligned}$$

Here, $\{(x_{tail_C},y_{tail_C})$, $(x_{tail_L},y_{tail_L})$, $(x_{tail_H},y_{tail_H})$, $(x_{tail_I},y_{tail_I})\}$ are the points along the curve of sperm flagellum of circular swimming, linear mean swimming, hyperactive, and immotile cells, respectively. Each flagellum consists of *M* points (typically $M=200$). The detailed placing of the sperm head ($\{(x_{H_C},y_{H_C})$, $(x_{H_L},y_{H_L})$, $(x_{H_H},y_{H_H})$, $(x_{H_I},y_{H_I})\}$) and the points along the curve of sperm flagellum ($\{(x_{tail_C},y_{tail_C})$, $(x_{tail_L},y_{tail_L})$, $(x_{tail_H},y_{tail_H})$, $(x_{tail_I},y_{tail_I})\}$) are provided below in the section titled “Swimming models”.

**Figure 4:**
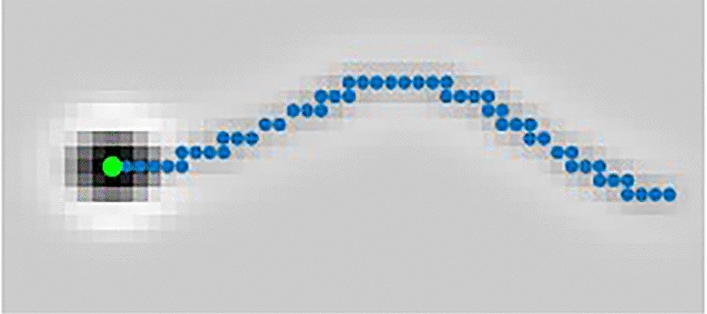
Simulated sperm image with points for the location of the head (green) and the curve of the flagellum (blue).

**Figure 5:**
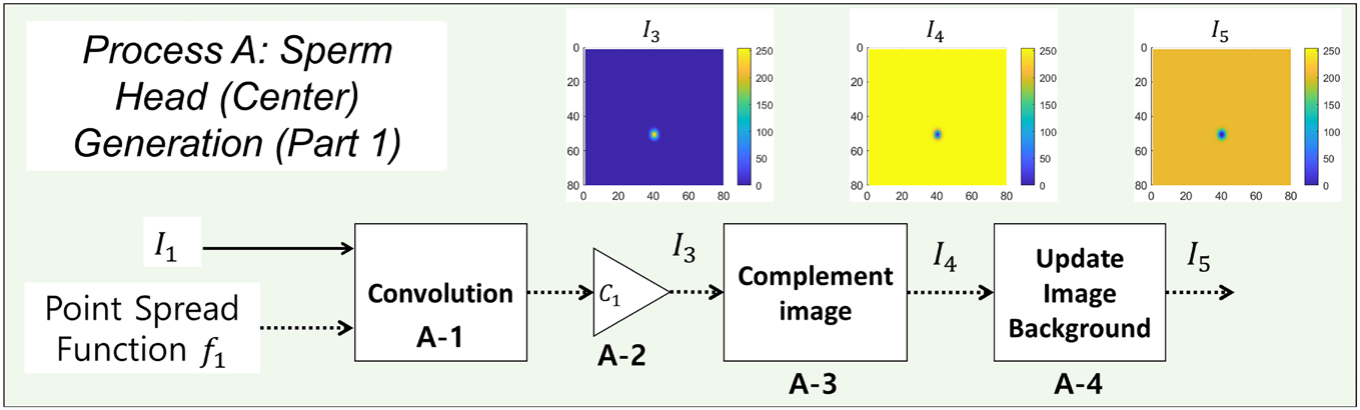
Flowchart of sperm head (center) image generation process. Image $I_1$ and point spread function $f_1$ are the inputs. The output is the simulated image of sperm head center $I_5$. $I_1$ and $f_1$ is convolved and then scaled by $C_1$ to generate $I_3$ (Processes A-1 and A-2). The image $I_3$ is complemented to generate the image $I_4$ (Process A-3). Lastly, the background is added to image $I_4$, generating an image of sperm head center $I_5$ (Process A-4).

An image of a simulated sperm cell is shown in Fig. 4 with the points for the location of sperm head (where $I_1(x,y) = 255$) and the curve of the flagellum (where $I_2(x,y) = 255$) shown in green and blue, respectively. In the simulation, grayscale values are used in the range between 0 to 255 (256 levels). In following sections, the five processes (A-E) are explained in detail. The flowcharts of processes (A-E) are given in Figs. 5, 7, 8, 9 and 10. Images of $I_3$-$I_9$ are accompanied by numbered colorbar that can be used to convert the image to grayscale.

#### Process A: sperm head (center) generation (Part 1)

**Figure 6:**
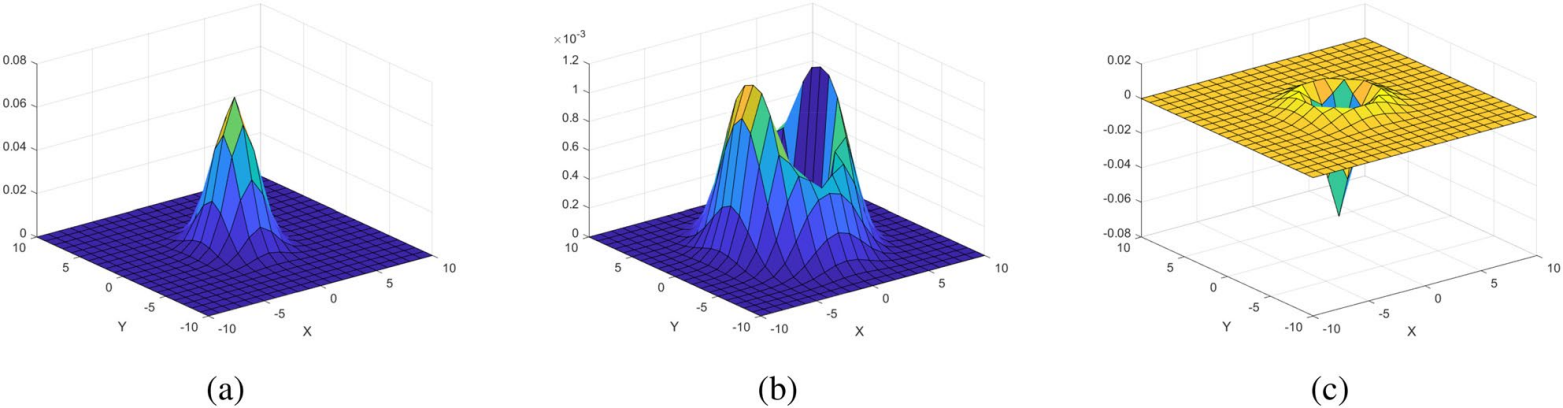
Point spread functions used in sperm cell image generation process; (**a**) $f_1$, (**b**) $f_2$, (**c**) $f_3$.

The flowchart of Process A is shown in Fig. 5. To generate the oval-like structure of the sperm head, image $I_1$ is convolved with a 2-D normal distribution filter $f_1$ (Process A-1); the filter is used as a point spread function (Ch. 3–5 in Gonzalez^39^). The point spread function $f_1$, a 2-D normal distribution, is defined as

3 $$\begin{aligned} f_1(x,y)=\frac{1}{2\pi \sigma _{x_G} \sigma _{y_G}}exp\left( -\left[ \frac{(\frac{x}{\sigma _{x_G}})^2+(\frac{y}{\sigma _{y_G}})^2}{2} \right] \right) . \end{aligned}$$

The 3-D surface plot of $f_1$ is shown in Fig. 6a. Standard deviations $\sigma _{x_G}$ and $\sigma _{y_G}$ control the length and width of the cell head. In the examples shown in this section, $\sigma _{x_G} = 1.86$ px and $\sigma _{y_G} = 2.86$ px. The size of the filter $f_1$ is $25\times 25$ px (*x* and *y* ranges from − 12 to 12). The resulting output image is then scaled by a constant $C_1$ to set the peak intensity value to be 255 and is denoted $I_3$ (Process A-2 in Fig. 5). Constant $C_1$ can be changed to control the intensity value of cell head. The image $I_3$ is complemented to produce the image of dark-oval-like center on a white background (Process A-3). In Process A-4, the background for the simulation image is added. Let $B_L$ be the background that will be added to the simulated image. The size of $B_L$ is *N* by *N*. In the examples in this section, a uniform background is assumed, where every value in the pixel is equal to 204 ($B_L(x,y) = 204$, 80% of 255). The value $255-B_L$ is subtracted from the image $I_4$ and any value below 0 is set to 0. The resulting image is denoted image $I_5$ ($I_5 = \max [0,I_4-(255-B_L)]$).

#### Process B: sperm head (membrane) generation (Part 2)

**Figure 7:**
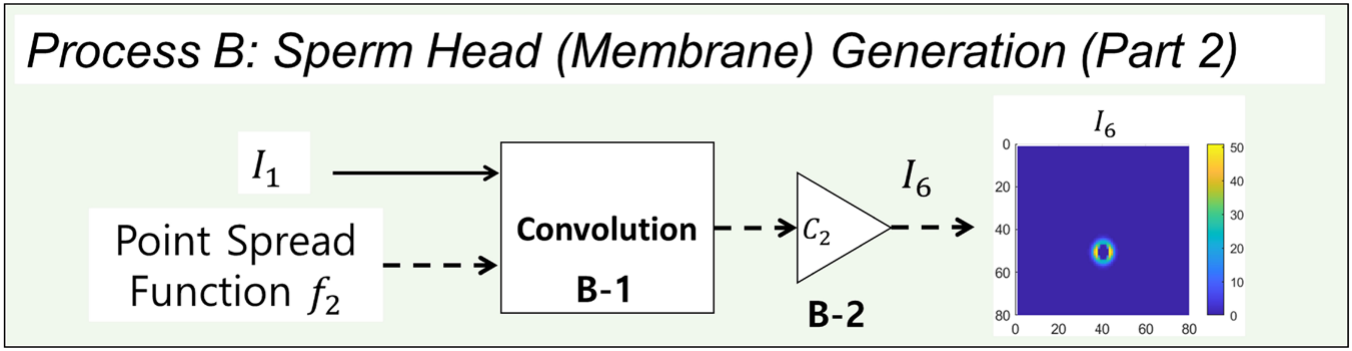
Flowchart of the image generation process of the sperm membrane. Image $I_1$ and point spread function $f_2$ are the inputs. The output is the simulated image of sperm membrane $I_6$. $I_1$ and $f_1$ is convolved and then scaled by $C_1$ to generate $I_6$ (Processes B-1 and B-2).

The flowchart of Process B is shown in Fig. 7. In typical semen image samples, one can observe a halo-like membrane surrounding the sperm head, as shown on the left of Fig. 1 (referred to as “a horseshoe-shaped halo” in Urbano et al.^3^). To generate this halo-like membrane around the cell, a modified version of Laplacian of the 2-D normal distribution filter is used as the second point spread function on image $I_1$. The Laplacian of the 2-D normal distribution filter is defined as

4 $$\begin{aligned} g(x,y)=\nabla ^2 \left( \frac{1}{2\pi \sigma _{x_L} \sigma _{y_L}}exp\left( -\left[ \frac{(\frac{x}{\sigma _{x_L}})^2+(\frac{y}{\sigma _{y_L}})^2}{2} \right] \right) \right) . \end{aligned}$$

The point spread function $f_2$ is defined as

5 $$\begin{aligned} f_2(x,y) = \max (0,g(x,y)). \end{aligned}$$

The resulting point spread function $f_2$ is shown in Fig. 6b. Standard deviations $\sigma _{x_L}$ and $\sigma _{y_L}$ control the length and width of the cell membrane. In the examples in this section, $\sigma _{x_L} = 2.79$ px and $\sigma _{y_L} = 4.29$ px. The size of the filter $f_2$ is $25\times 25$ px (*x* and *y* ranges from − 12 to 12). The point spread function $f_2$ is convolved with image $I_1$, generating the image of a membrane (Process B-1). The resulting output image is then scaled by constant $C_2$ (Process B-2) to set the peak intensity to be 51 (peak intensity value of cell membrane, 20% of 255). This image of the membrane is denoted $I_6$. Constant $C_2$ can be changed to control the intensity value of the cell membrane.

**Figure 8:**
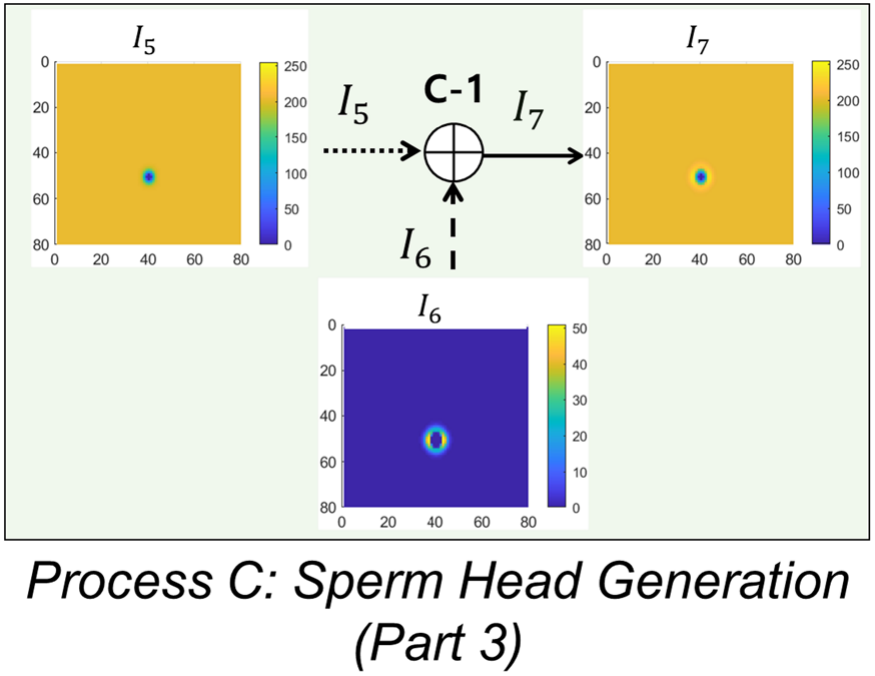
Diagram of sperm head image merging process. Images $I_5$ and $I_6$ are the inputs. The output is the simulated image of sperm head $I_7$. $I_5$ and $I_6$ are added to generate $I_7$ (Process C-1).

#### Process C: sperm head generation (Part 3)

The flowchart of Process C is shown in Fig. 8. In Process C, the images of the sperm head center $I_5$ and the image of the flagellum $I_6$ are added to complete the image of sperm head. The resulting sperm head image is shown in Fig. 8 and is denoted $I_7$.

**Figure 9:**
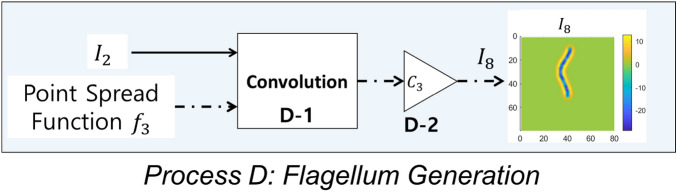
Flowchart of flagellum generation process. Image $I_2$ and point spread function $f_3$ are the inputs. The output is the simulated image of flagellum $I_7$. $I_2$ and $f_3$ are convolved and then scaled by $C_3$ to generate $I_7$ (Processes D-1 and D-2).

#### Process D: sperm flagellum generation

The flowchart of Process D is shown in Fig. 9. To generate the flagellum, image $I_2$ (Eq. 2) is convolved with point spread function $f_3$ (Eq. 6) to generate the image of uniform calibre look of the flagellum. The point spread function $f_3$ is defined as the Laplacian of Gaussian filter (equal to the Eq. (4) with $\sigma _{x_L}=\sigma _{y_L}=\sigma _f$), namely

6 $$\begin{aligned} f_3(x,y)=\nabla ^2 \left( \frac{1}{2\pi \sigma _{f}^2}exp\left( -\left[ \frac{(\frac{x}{\sigma _{f}})^2+(\frac{y}{\sigma _{f}})^2}{2} \right] \right) \right) . \end{aligned}$$

The 3-D surface plot of $f_3$ is shown in Fig. 6c. Standard deviation $\sigma _{f}$ controls the width of the flagellum. In the examples in this section, $\sigma _{f} = 1.5$ px. The size of the filter $f_3$ is $25\times 25$ px (*x* and *y* ranges from -12 to 12). The output image is then scaled by constant $C_3$ to set the peak intensity of the membrane as 13 (approximately 5% of 255). Constant $C_3$ can be changed to control the intensity of the flagellum. The resulting image of the flagellum is a long membrane-like curve. The scaled image of the flagellum is denoted $I_8$.

**Figure 10:**
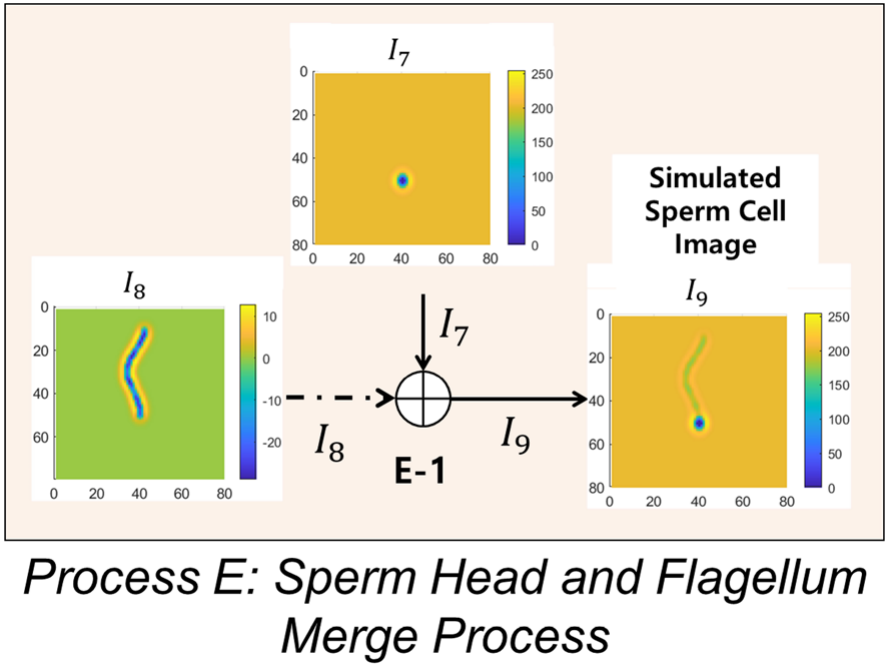
Diagram of sperm head and flagellum image merging process. Images $I_7$ and $I_8$ are the inputs. The output is the simulated image of sperm cell $I_9$. $I_7$ and $I_8$ are added to generate $I_9$ (Process E-1).

#### Process E: sperm head and flagellum merge

Finally, in Process E, the image of cell head, $I_7$, and the image of the flagellum, $I_8$, are added to generate the image of a sperm cell ($I_9$ in Figs. 3 and 10). Process E is shown in Fig. 10.

**Figure 11:**
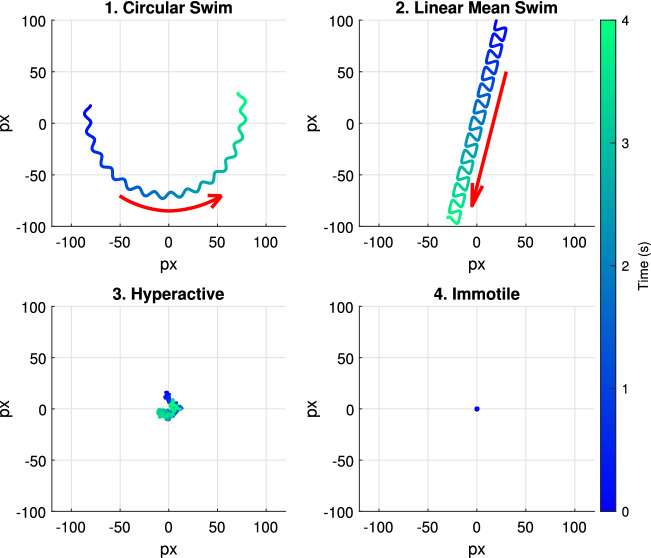
Simulated swimming path of the four swimming modes: circular swim, linear mean swim, hyperactive, and immotile. The color bar indicates the color of a track with respect to time. The duration of movement is 4 s.

### Swimming models

In this section, we describe the swim/movement models of the head and the flagellum of a sperm cell. The swimming models describe how the position of the head and the flagellum change over time. These positions define the points used to generate $I_1$ (cell head, Eq. (1)) and $I_2$ (cell flagellum, Eq. 2).

We categorize the sperm movement into the following four swimming modes:

1. Circular swim,
2. Linear mean swim,
3. Hyperactivated,
4. Immotile, or dead.

Figure 11 shows simulated tracks for the four swimming modes (4 seconds long). The tracks indicate the location of the head of a cell and the arrow indicates the direction of movement.

Circular swimming cells and linear mean swimming cells actively propel themselves forward, either on a large circular or linear path. Examples of (1) circular swim and (2) linear mean swim are shown on top of Fig. 11. The red arrow indicates the direction of movement for the cell. (3) Hyperactivated sperm cells do not travel significantly away from their initial location. The movements of hyperactive cells are described as “vigorous”, “whiplash type”, or “frantic”^40,41^. An example is shown in bottom left of Fig. 11. (4) Immotile movements define cells which show barely any movement at all (shown in bottom right of Fig. 11).

We synthesized the models to describe circular and linear mean movements based on the sperm tracks recorded in the literature^3,8,29,33,42^. Hyperactive swim, described to be “frantic” and “vigorous” is modeled through as Brownian motion to reflect the randomness in the cell movement^43^. In following sections, we describe each movement in detail.

**Table 1:** Simulation of a circular swimming cell.

Circular swim model—head	$x_{H_C}(t)=(r_c+a\sin (2\pi f_st))\cos (2\pi f_ct) + C_{x_c}$	(7a)
	$$y_{H_C}(t)=(r_c+a\sin (2\pi f_st))\sin (2\pi f_ct) + C_{y_c}$$	(7a)
Circular swim model—flagellum	$x_{T_c}(k,t) = b(k)\sin \left[ 2\pi \left( \frac{k}{M}-f_st\right) \right]$	(8a)
	$$y_{T_c}(k) = -\lambda \frac{k}{M}$$	(8b)
	$$\begin{bmatrix} x_{tail_{C}}\\ y_{tail_{C}} \end{bmatrix}= R(2\pi f_s t) \begin{bmatrix} x_{T_c}\\ y_{T_c} \end{bmatrix} + \begin{bmatrix} x_{H_C}\\ y_{H_C} \end{bmatrix}$$	(9)
	$$R(\cdot ) = \begin{bmatrix} \cos (\cdot ) &{} -\sin (\cdot )\\ \sin (\cdot ) &{} \cos (\cdot ) \end{bmatrix}$$	(10)
	$$b(k) = a\left( \alpha \frac{\lambda k}{M}+\beta \right) , \; \alpha = 0.02, \; \beta = 0.8$$	(11)

$(x_{H_C},y_{H_C}):$ head position of circular swimming cell.

$r_{c}:$ radius of the circular path.

*a* :  amplitude of the sinusoid modulated on the circular path.

$f_{s}:$ frequency of the sinusoid modulated on the circular path (Hz).

$f_{c}:$ frequency of the circular cycle (cycle/s).

$(C_{x_c},C_{y_c}):$ vertical and horizontal offset constant.

$(x_{Tail_C},y_{Tail_C}):$ a set of *k* points along the center of the flagellum of circular swimming cell.

$\lambda :$ wavelength of flagellum (distance between the start and the end of the flagellum).

$$R(\cdot ):$$ rotation matrix.

$b(\cdot ):$ local variation in beating amplitude along the flagellum.

#### Circular swim model

**Figure 12:**
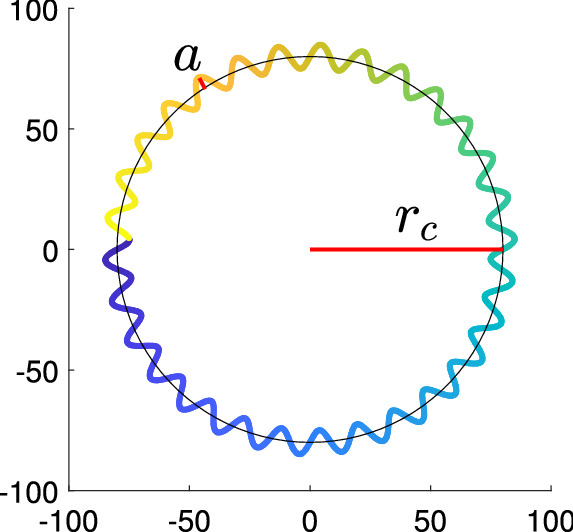
Trajectory of simulated circular swimming cell.

A sperm cell exhibiting a circular swim moves along a circular path with oscillations about this path caused by the beatings of the flagellum^33^. This circular swim can be represented as a sinusoidal modulated circular path (Fig. 12). The equations used to model the movement of the head and the flagellum of a circular swimming cell are given in Table 1. The 2-D position of the cell’s head is determined by Eqs. (7a), (7b). $r_c$ is the radius of the overall circular path, $f_s$ is the frequency of the sinusoid modulated on the circular path (Hz), *a* is the amplitude of the sinusoid modulated on the circular path, and $f_c$ is the frequency of the circular cycle (cycle/sec). $C_{x_c}$ and $C_{y_c}$ are horizontal and vertical offsets, respectively.

$(x_{T_c}(k,t),y_{T_c}(k))$ are Cartesian coordinates of the points along the center curve of the flagellum with $k = 1,2,3,\ldots ,M$, where in the default setting we used $M = 200$. The flagellum of circular swimming cells follows Dresdner’s model of sperm flagellum (Eq. 8a)^24^. Equation (8a) determines the oscillation of the flagellum ($x_{T_c}$), and Eq. (8b) determines the end to end length (wavelength $\lambda$) of the flagellum (distance between the start and the end of the flagellum). *b*(*k*) is the local variation in beating amplitude along the flagellum. The points $(x_{T_c},y_{T_c})$ are rotated using the rotation matrix $R(\cdot )$ to match the direction of movement, and shifted to the location of the sperm head ($x_{H_C},y_{H_C}$) (Eq. 9); the points of the flagellum of a circular swimming cell are denoted $(x_{tail_C},y_{tail_C})$. In the examples shown in this paper, the local variation in the beating amplitude, *b*(*k*), follows the affine function $b(k) = a\left( \alpha \frac{\lambda k}{M}+\beta \right) ,$ with $\alpha = 0.02, \; \beta = 0.8$ for circular swimming cell. Here, *a* is the amplitude of a sinusoid modulated on the circular path, and $\lambda$ is the wavelength of the flagellum.

**Figure 13:**
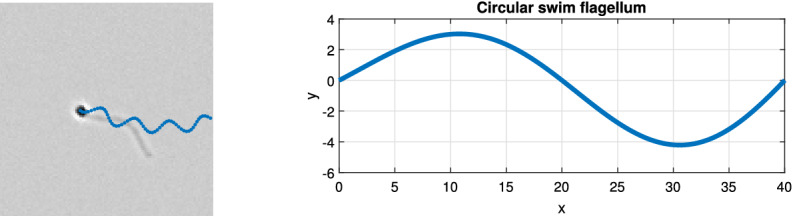
(Left) Simulated image of circular swimming cell with past track shown in blue. (Right) Plot of the flagellum of circular swimming cell. In this example, wavelength ($\lambda$) is 40 pixels and amplitude (*a*) is 4 pixels.

An example of simulated flagellum of a circular swimming cell is shown in Fig. 13. On the left is a simulated image of a circular swimming cell with the path shown in blue line. On the right is a curve which represents the flagellum of that cell. The example in this image uses amplitude of $a = 4$ pixels and wavelength of $\lambda = 40$ pixels.

**Table 2:** Simulation of a linear mean swimming cell.

Linear mean swim model—head	$$\begin{bmatrix} x_{c}\\ y_{c} \end{bmatrix} = \begin{bmatrix} V\cos (\theta _{r})t+C_{x_L}\\ V\sin (\theta _{r})t+C_{y_L} \end{bmatrix}$$	(12)
	$$P =\begin{bmatrix} P_x(t)\\ P_y(t) \end{bmatrix} =\begin{bmatrix} \frac{r_{v}}{2}\sin (4\pi f_{l}t)\\ \frac{r_{h}A_c}{2}[\sin (2\pi f_{l}t)+A_{har}\sin (6\pi f_{l}t)] \end{bmatrix}$$	(13)
	$$A_c = \frac{1}{\max \limits _{\theta } (\sin (\theta )+A_{har}\sin (3\theta ))}$$	(14)
	$$\begin{bmatrix} x_{H_L}\\ y_{H_L} \end{bmatrix} =R(\theta _r)P +\begin{bmatrix} x_{c}\\ y_{c} \end{bmatrix}$$	(15)
Linear mean swim model—flagellum	$$\begin{bmatrix} x_{o}(k,t)\\ y_{o}(k,t) \end{bmatrix} =\begin{bmatrix} b_1(k)x_{T}(k,t) \\ b_2(k)y_{T}(k,t) \end{bmatrix}$$	(16)
	$$x_{T}(k,t) = \frac{r_{v}}{2} \sin \left( 4\pi \left( \frac{k}{M}+f_{l}t\right) \right)$$	(17a)
	$$y_{T}(k,t) = \frac{r_{h}}{2} \sin \left( 2\pi \left( \frac{k}{M}+f_{l}t\right) \right)$$	(17b)
	$$\begin{bmatrix} x_{LM}(k,t)\\ y_{LM}(k,t) \end{bmatrix} =\begin{bmatrix} x_{o}(k,t)\\ y_{o}(k,t) \end{bmatrix} -\begin{bmatrix} \frac{\lambda k}{M} \\ 0 \end{bmatrix}$$	(18)
	$$y_{LM_2}(k,t) = y_{LM}(k,t) - [P_y(t) - y_T(0,t)]b_3(\psi )$$	(19)
	$$\begin{bmatrix} x_{tail_{LM}}\\ y_{tail_{LM}} \end{bmatrix}= R(\theta _r) \begin{bmatrix} x_{LM}\\ y_{LM_2} \end{bmatrix} + \begin{bmatrix} x_{H_L}\\ y_{H_L} \end{bmatrix}$$	(20)
	$$b_1(k) = \frac{1}{1+e^{(\alpha k/M + \beta )}}$$	(21a)
	$$b_2(k) = e^{-\gamma _1 k/M}$$	(21b)
	$$b_3(k) = 1-e^{-\gamma _2 k/M}$$	(21c)

$(x_{H_L},y_{H_L}):$ position of the sperm head of linear swimming cell .

$(C_{x_L},C_{y_L}):$ horizontal and vertical offset constant (pixels).

*V* :  straight line path velocity (pixels/sec).

$f_l:$ rate of change in ribbon angle (Hz).

$r_h, r_v:$ width and height of ribbon (pixels).

$A_{har}:$ user defined ratio between the first and the third harmonics.

$A_{c}:$ correction constant for defined width of the ribbon.

$\theta _r:$ direction of the forward movement (radian).

$(x_{Tail_{LM}},y_{Tail_{LM}}):$ a set of *k* points along the center of the flagellum of linear mean swimming cell.

$b_1(k), b_2(k):$ local horizontal and vertical variation in beating amplitude along the flagellum.

$b_3(k):$ flagellum position correction function.

#### Linear mean swim model

In linear mean swim, a sperm cell moves along a straight-line path, rolling side to side as it propels itself forward. This feature causes a ribbon-like movement about the straight line. The equations used to model the movement of the head and the flagellum of linear mean swimming cell are given in Table 2.

**Figure 14:**
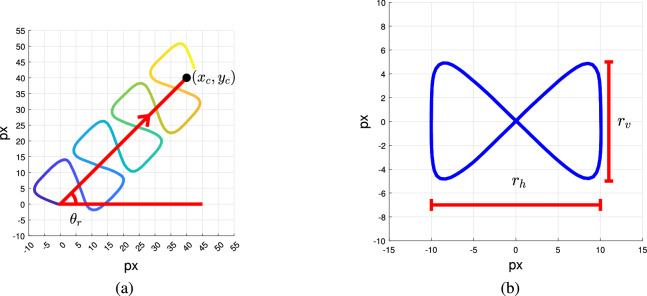
(**a**) Trajectory of simulated linear mean swimming cell (Eqs. 12–15). (**b**) Ribbon-like oscillatory movement along the straight-line path of linear mean swimming cells (Eq. 13).

We generate the cell head movement (ribbon-like movement along a linear path) using Eqs. (12–15). The position along the linear path is denoted ($x_c,y_c$) and the position along the ribbon-like movement is denoted ($P_x(t),P_y(t)$). The resulting path is shown in Fig.  14a.

The straight-line path is a linear function of time *t* with initial positions of $(C_{x_L}, C_{y_L})$. The straight-line path is a line segment from initial positions $(C_{x_L}, C_{y_L})$ to the point ($x_c,y_c$) (as shown in Fig. 14). The horizontal and vertical positions along the linear path ($x_c,y_c$) change at the rate of horizontal and vertical velocity in the direction of forward movement $\theta _r$ (Eq. 12). The position along a “ribbon-like” path ($P_x(t),P_y(t)$) is generated using Eqs. (13–14). The ribbon is shown in Fig. 14b, where $r_v$ is the height of the ribbon and $r_h$ is its width. $P_x(t)$ and $P_y(t)$ are periodic functions of time *t*. $P_x(t)$ is a sinusoid with a frequency of $2f_l$ and amplitude of $\frac{r_v}{2}$. $P_y(t)$ is a sum of two sinusoids of frequencies of $f_l$ and $3f_l$ and amplitudes of $\frac{r_h A_c}{2}$ and $A_{har}$, respectively. $A_c$ is a correction constant to set the amplitude of function $P_y(t)$ as $\frac{r_h}{2}$ (Eq. 14). The resulting ribbon-like path is shown in Fig. 14b. $A_{har}$ is the ratio between the two sinusoids of function $P_y(t)$. The examples in this paper use $A_{har}=0.1$.

The position of the cell along the ribbon, ($P_x(t),P_y(t)$), is multiplied by rotation matrix $R(\cdot )$ resulting in a rotation by $\theta _r$ (Eq. (15), $R(\theta _r)P$). Lastly, The position on the ribbon-like path $R(\theta _r)P$ is added to the position along the straight-line path $(x_c,y_c)$ to define the position of the sperm head at all times (Eq. 15).

**Figure 15:**
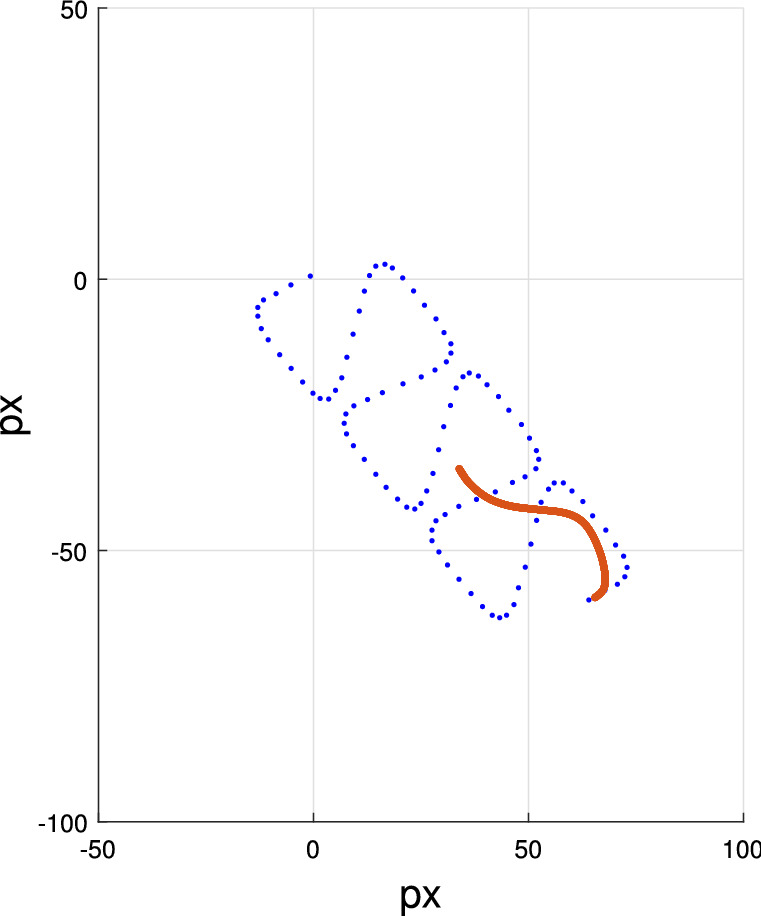
Example of sperm flagellum generation for linear mean swimming cell. (Orange) Simulated flagellum. (Blue) Track of linear mean swimming cell.

The flagellum is generated using Eqs. (16–21). Dresdner’s model is used to define the horizontal and vertical oscillations in the flagellum ($x_o(k,t),y_o(k,t)$). $x_o(k,t)$ and $y_o(k,t)$ are discrete set of points for $k = 1,2,3,\ldots ,M$ realized at time *t*. The value of *M* in our simulation is 200. An example of simulated flagellum of linear mean swimming cell is shown in Fig. 15. The blue dots represent the successive location of the sperm head. We also show, for one of these locations, the associated flagellum in orange. We use the fundamental frequency of the ribbon in horizontal ($2f_l$ for $P_x(t)$) and vertical directions ($f_l$ for $P_y(t)$) to determine the movement of the flagellum (Eqs. (13) and (17)). The local variation in beating amplitude for $x_{T}(k,t)$ and $y_{T}(k,t)$ are $b_1(\psi )$ and $b_2(k)$, respectively (Eqs. (21a) and (21b)).

The function $b_1(k)$ (Eq. (21a)) is a transformed sigmoid function, where the values of $\alpha$ and $\beta$ determine the shift and the compression/expansion of the function; $b_2(k)$ (Eq. 21b) is a decaying exponential function. Together, these functions provide the desirable realistic visual effect.

We define a line segment from the head to the end of the flagellum ($f(k) = -\frac{\lambda k}{M}, \; k = 1,2,3,\ldots ,M$). The oscillations $x_{o}(k,t)$ and $y_{o}(k,t)$ are added to this straight line (Eq. 18).

The differences in the vertical location of the head $P_y(t)$ and the vertical position of the flagellum $y_{T}(k,t)$ cause offset in placement of the flagellum because we have only considered the fundamental frequency component, $f_l$, of $P_y(t)$ to generate ($y_{T}(k,t)$). This mismatch is corrected by Eqs. (19) and (21c).

Lastly, the flagellum is rotated to match the direction of movement and shifted to the position of the cell head (Eq. 20). The constants in Eqs. (21a), (21b), and (21c) in the example of we provide here were set to: $\alpha = 22, \beta = -2, \gamma _1 = 5, \gamma _2 = 1.5$.

**Table 3:** Simulation of a hyperactive swimming cell.

Hyperactive swim model—head	$x(t) = \mu _x t + \sigma _{x_b} W(t) \text { and}$	(22a)
	$$y(t) = \mu _y t + \sigma _{y_b} W(t)$$	(22b)
	$$x_{H_H}(t) = x_{H_H}(t-T) + \sigma _{b} W(T) \text { and}$$	(23a)
	$$y_{H_H}(t) = y_{H_H}(t-T) + \sigma _{b} W(T),$$	(23b)
Hyperactive swim model - flagellum	$(x_{tail_H}(k,t), y_{tail_H}(k,t)) =$ $\text {Linear interpolation on a set of points}$ $[(x_{H_L}(0), y_{H_L}(0)), (x_{H_L}(1), y_{H_L}(1)),$ $(x_{H_L}(2), y_{H_L}(2)), \ldots , (x_{H_L}(n), y_{H_L}(n))]$ $\text {where} \; x_{H_L}(n) = x_{H_H}(t-nT),$ $\qquad \; \; \, y_{H_L}(n) = y_{H_H}(t-nT)$	(24)

$(\mu _x, \mu _y):$ drift coefficients of Brownian motion.

$(\sigma _{x_b}, \sigma _{y_b}):$ diffusion coefficients of Brownian motion.

*W*(*t*) :  standard 1-dimensional Brownian motion ($W(t) \sim N(0,t)$).

$(x_{H_H}, y_{H_H}):$ position of the sperm head of hyperactive cell.

*T* :  the difference in time between each simulation frame.

$(x_{Tail_H},y_{Tail_H}):$ a curve along the center of the flagellum of hyperactive cell.

#### Hyperactive swim model

The equations used to model the movement of the head and the flagellum of hyperactive swimming cell are given in Table 3. For each dimension (x and y), the movement of a cell follows Eq. (22), where ($\mu _x, \mu _y)$ are drift coefficients of Brownian motion, ($\sigma _{x_b}, \sigma _{y_b}$) are diffusion coefficients of Brownian motion, and *W*(*t*) is the standard 1-dimensional Brownian motion ($W(t) \sim N(0,t)$). Our simulation assumed no drift ($\mu _x = \mu _y = 0$), and the diffusion coefficients are assumed to be the same for the horizontal (x) and the vertical (y) directions ($\sigma _{x_b}^2 = \sigma _{y_b}^2 = \sigma _{b}^2$). The location of the sperm head of hyperactive cell is defined by Eq. (23), where *T* is the difference in time between each simulation frame ($\frac{sec}{\text {frame}}$).

The flagellum of the hyperactive swimming cell is defined to be the linear interpolation on a set of points $[(x_{H_L}(t),y_{H_L}(t)),(x_{H_L}(t-T),y_{H_L}(t-T)),(x_{H_L}(t-2T),y_{H_L}(t-2T)),\ldots ,(x_{H_L}(t-nT),y_{H_L}(t-nT))]$ (a set of location of the cell from time $t-nT$ to *t*, Eq. (24)). In our simulations, the value *n* is set to $\frac{0.2 sec}{T}$ or rounded up to the nearest integer value.

**Table 4:** Simulation of an immotile cell.

Immotile cell model—head	$x_{H_I}(t) = x_{H_I}(0) \text { and}$	(25a)
	$$y_{H_I}(t) = y_{H_I}(0)$$	(25b)
Immotile cell model - flagellum	$(x_{tail_I}(k,t), y_{tail_I}(k,t)) =$ $\text {Linear interpolation on a set of points}$ $[(x_{I_L}(0), y_{I_L}(0)), (x_{I_L}(1), y_{I_L}(1)),$ $(x_{I_L}(2), y_{I_L}(2)), \ldots , (x_{I_L}(n), y_{I_L}(n))]$ $\text {where} \; x_{I_L}(n) = x_{H_H}(-nT),$ $\qquad \; \; \, y_{I_L}(n) = y_{H_H}(-nT)$	(26)

$(x_{H_I}, y_{H_I}):$ position of the sperm head of immotile cell.

$(x_{Tail_I},y_{Tail_I}):$ a curve along the center of the flagellum of immotile cell.

#### Immotile or dead cell model

Immotile or dead cells are simulated as non-moving objects. The equations used to model the movement of the head and the flagellum of immotile cell are given in Table 4.

Throughout the simulation, the head and the flagellum of the cell will not move (Eqs. 25, 26). The flagellum is generated by taking a snapshot of the flagellum of a hyperactive cell. The flagellum of the immotile cell is generated using Eq. (26).

**Figure 16:**
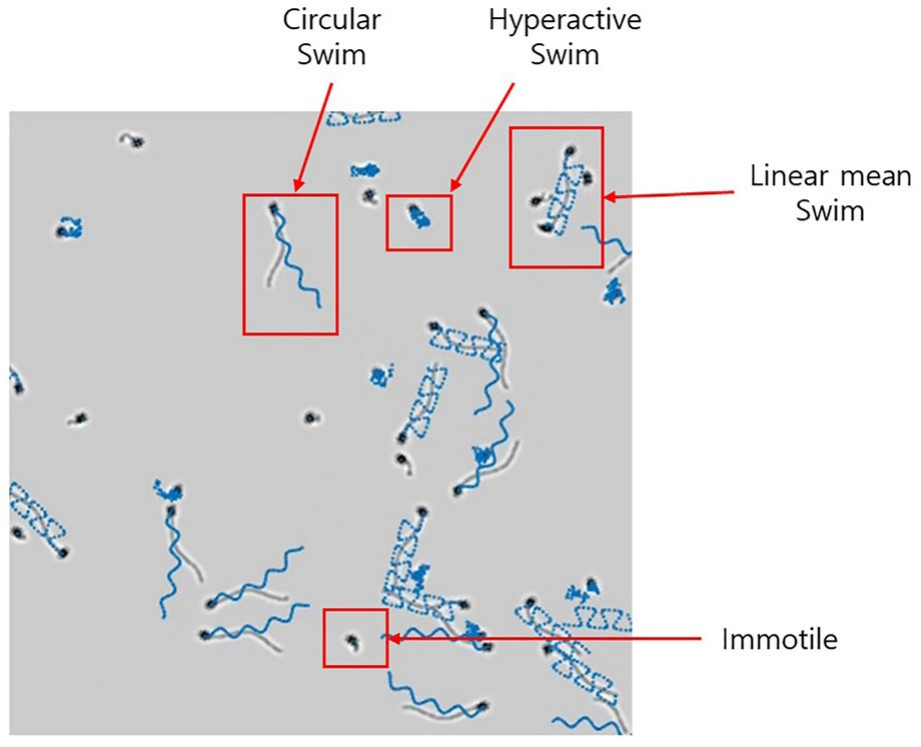
Example of simulated image with track of each cell shown in blue.

In Fig. 16, an example the simulated semen sample is shown, where the cells of four different swimming modes can be seen. The trajectories of each cell are shown in blue line. In the simulation software (available in Choi et al.^34^), parameters for cell concentration, appearance, and swimming modes can be changed to generate the user’s desired semen image for testing.

**Figure 17:**
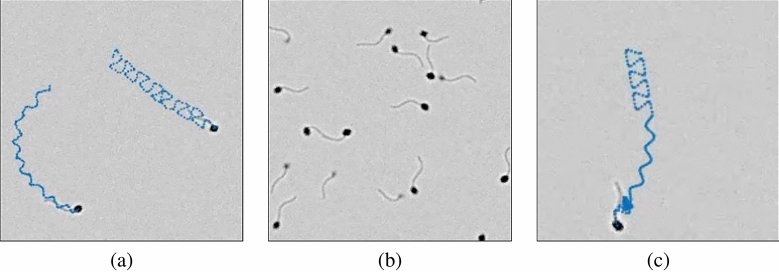
(**a**) Simulation image of two cells with additive random noise; tracks of the two cells shown in blue. The random noise in this example is Gaussian random variable. (**b**) Simulation image of sperm cells with variable intensity. (**c**) Simulation image of a sperm cell making transition to other swimming modes.

In Fig. 17, we show 3 additional scenarios which are simulation features. These simulation features are used to generate dynamic scenarios for testing of CASA algorithms and systems.

The first feature adds noise to the position of each sperm cell (example shown in Fig. 17a). The addition of noise is used to mimic the random movements caused by the cell and the surrounding fluid.

The second feature assigns a different intensity to each sperm cell (example shown in Fig. 17b). This feature is to simulate an environment where the sperm cells appear differently due to the depth of the chamber or slide used to observe the semen sample. The chamber that is used to observe semen is usually 10 to $20\; \upmu$m deep^38^. Certain cells may be swimming much deeper within the chamber when other cells may be swimming close to the top of the chamber. This causes some cells to look faded. The second feature is used to mimic such scenario.

Sperm cells change their swimming mode as time progresses^32^. The third feature assigns transition probabilities to define how likely a cell will change from one swimming mode to the other. In the example shown in Fig. 17c, a sperm cell goes from linear mean swim to circular swim to hyperactive swim. As an example, one can assign probability that a cell undergoing linear mean swim has 85% chance of staying in linear mean swim, 10% chance of transitioning to circular swim, and 5% chance of transitioning to hyperactive swim after 1 second (0% chance to change into immotile). In Choi et al.^34^, we provide sample images generated using these three different functionalities as examples.

## Testing setup

For testing, we simulated images based on real semen image samples. The semen samples used in the study were collected by us for previous studies^3,4^ and are available in the public domain^35^. The images of the samples were provided by the In-Vitro Fertilization laboratories at Penn Fertility Care. Each specimen was allowed to liquefy for 30–40 minutes at room temperature and was washed in media. The washed semen samples were pipetted on a 20 $\upmu$m deep Vitrolife MicroCell chamber for data collection. For detailed explanation on the preparation process of the samples, please refer to Urbano et al.^3^ and Urbano^4^.

The parameters we have extracted from the real images were *the image background* $B_L$, *the noise variance* $\sigma _N^2$, *the size of sperm head*, *the number of cells in the image* $N_C$, and *the number of non-moving cells* $N_D$. The values of the parameters are given in Table 5. The sperm cells in the image were labeled manually. The process used to calculate these parameters are provided in the additional file [see Additional file 1].

**Table 5:** Simulation parameters for segmentation and localization testing.

	Noise variance $\sigma _N^2$	Radius $r_M$ (major axis)	Radius $r_m$ (minor axis)	Number of cells $N_C$	Number of non-moving cells $N_D$
Sample 1	$8.22 \times 10^{-6}$	2.86 px	1.86 px	10	3

## Testing

Two use cases of the simulation were explored. First, we tested five sperm cell detection algorithms and assessed their performances. The cell detection algorithms consists of two major components, which are segmentation and localization^39^. In segmentation, regions in the image that contains sperm cells are separated from the image. In localization, the locations of sperm cells in the segmented regions are labeled. The performance of segmentation and localization was evaluated in terms of optimal subpattern assignment (OSPA) distance ($c = 20,\; p = 2$)^44^, precision and recall rates^45^. OSPA distance is a metric that quantifies errors in distance between the ground truth and detection and in cardinality (difference in number between ground truths and detections). Precision is the ratio between true positives (true matches) to the total number of detections. Recall is the ratio between true positives to total number of ground truth. Ideally, the value of OSPA distance is 0 and the values of precision and recall are 1. The calculation of OSPA distance, precision, and recall in real samples was made possible by manual labeling of sperm locations.

Second, we tested four tracking algorithms for sperm tracking (nearest neighbor (NN), global nearest neighbor (GNN), probabilistic data association filter (PDAF), and joint probabilistic data association filter (JPDAF))^46^. The purpose of a tracking algorithm is to track each of the sperm cells as time progresses. The calculated tracks are then used to provide parameters that describe the motility of sperm cells in the semen sample (e.g., percent of motile cells, velocity of sperm cells). The performance of the tracking algorithms was assessed in terms of Multiple Object Tracking Precision (MOTP) and Multiple Object Tracking Accuracy (MOTA) with cutoff distance of $c_{T} = 5$ px^47^. MOTP is the sum of the distance between the matched ground truth and detected tracks. MOTA is a metric that quantifies the error in false positive ${\overline{FP}}$ (false alarm), false negative ${\overline{M}}$ (missed detection), and track mismatch ${\overline{MME}}$. The MOTA is defined to be $MOTA = 1 - ({\overline{FP}} + {\overline{M}} + {\overline{MME}})$. Ideally the value of MOTP is zero and MOTA is 1 (${\overline{FP}} = {\overline{M}} = {\overline{MME}} = 0$). Detailed explanation of the assessment metrics is given in the additional file [see Additional file 1].

### Applying the simulation to assess segmentation, localization, and tracking

The real semen image and the simulated images were tested using five (5) different algorithms for detection (segmentation and localization). These algorithms were the following:

(Otsu) Binarization of image using Otsu’s thresholding followed by morphological enhancements (closing, dilation, and erosion)^48^.
(Adaptive) Binarization of image using the adaptive thresholding method of Bradley^49^ with the sensitivity defined to be 0.8, followed by morphological enhancements (closing, dilation, and erosion).
(Spot-enhancement) Binarization of spot-enhanced image using Otsu’s thresholding, followed by morphological enhancements (closing, dilation, and erosion) (method proposed in Urbano et al.^3^).
(Edge-detection) Edge detection of median-filtered image using Sobel operator (modified algorithm proposed in Abbiramy and Shanthi (2010)^5^) followed by morphological enhancements (dilation, closing, and erosion).
(GMM) Motion detection algorithm using Mixture of Gaussian Model (GMM) with the number of training frames set to 20, number of Gaussian modes in the mixture model set to 3, learning rate set to 0.005, and background ratio set to 0.7^11,50^ followed by morphological enhancements (closing, dilation, and erosion).

**Figure 18:**
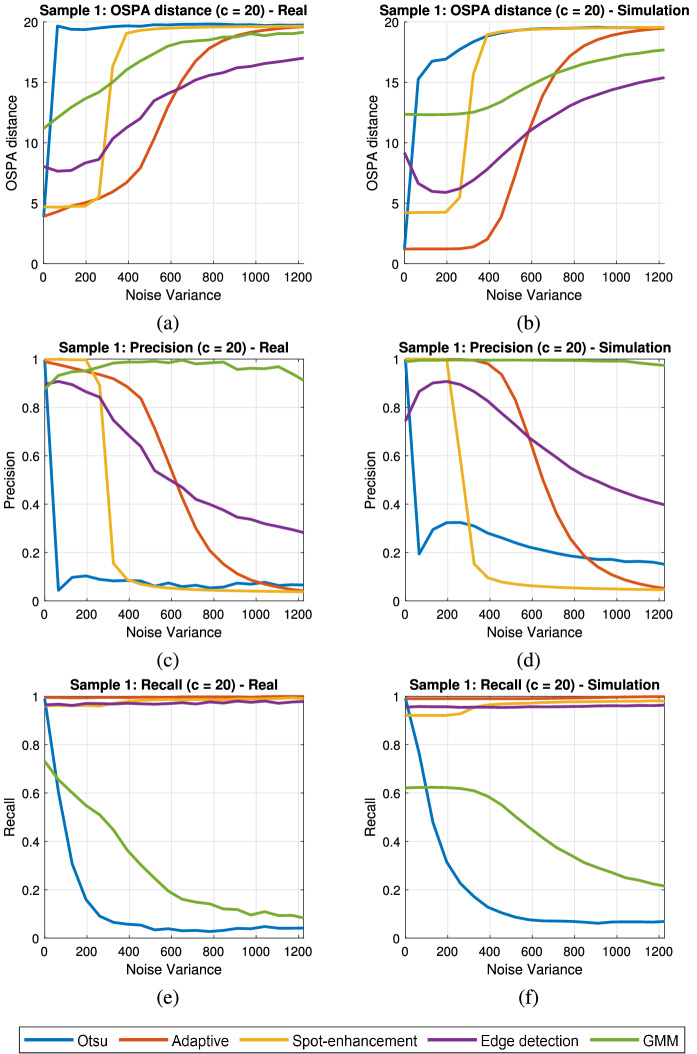
OSPA distance, precision and recall rates of sample 1 for varying levels of additive Gaussian noise (**a**,**c**,**e**) real, (**b**,**d**,**f**) simulation.

A total of 20 different simulated images were generated for testing using the parameters obtained from a real human sample (sample 1). The size of the frame was $250 \times 250$ px. In the simulated images, the non-moving cells were modeled as immotile cells. The moving cells ($N_C - N_D$) were modeled as either linear mean swimming ($50\%$) or circular swimming ($50\%$) cells. The algorithms were tested on real and simulated images. To each one of the images (real and simulated), we added zero-mean Gaussian noise with variance ranging from 0 to 1225 (standard deviation of 35 in grayscale). Figure 18 shows the OSPA distances, precision, and recall rates for varying levels of noise of real and simulated images.

The graphs show that the Adaptive algorithm exhibits the best performance in terms of OSPA (smallest distance away from ground truth) under low noise levels (noise variance approximately below 600), followed by the Spot-enhancement algorithm, the Edge-detection algorithm, the GMM algorithm, and the Otsu algorithm, respectively (Fig. 18a,b).

In terms of precision, sharp loss in performance is observed for the Otsu algorithm and the Spot-enhancement algorithm when the noise level increases beyond the noise variance of 100 and 300, respectively. Precision for the Adaptive algorithm and the Edge-detection algorithm also decreases as the noise level increases, where the Edge-detection algorithm becomes more robust than Adaptive algorithm in high levels of noise (approximately above noise variance of 600). The GMM algorithm shows the highest level of robustness, having almost no degradation in performance in precision (small number of false alarms).

In terms of recall, almost no change is observed between the noise variance of 0–1225 for the Adaptive algorithm, the Spot-enhancement algorithm, and the Edge-detection algorithm. The Otsu algorithm and the GMM algorithm showed degradation in performance when noise level increased, the Otsu algorithm showing sharp decline around noise variance of 100 and the GMM algorithm showing gradual decline between noise variance value of 0–1225.

The OSPA distance, precision and recall rates of the five different algorithms show similar trends for the real sample image and its simulated image. Overall, the Adaptive algorithm performed best in low level of noise (below noise variance of 600) and the Edge-detection algorithm has performed the best in high level of noise (above noise variance of 600). The Otsu algorithm showed the worst performance. Spot-enhancement algorithm performed well in low levels of noise, but failed when the noise level rose above noise variance of 300. GMM algorithm was the best algorithm in terms of precision, however did not show good performance in terms of OSPA distance and recall. Additional results for another real human sample (sample 2) are provided in the additional file [see Additional file 1].

### Applying the simulation to assess tracking

Four (4) tracking algorithms (NN, GNN, PDAF, and JPDAF) were tested on simulated images. The codes for tracking algorithms were written by Leonardo Urbano and are available in Urbano et al.^51^. The output of each tracking algorithm is the track information of all the detected cells in the semen image.

We evaluated the performance of NN, GNN, PDAF, and JPDAF algorithms for varying numbers of cells. Each simulated image consisted of 20, 40, 100, or 200 cells. Each cell in each image was assigned to a swim type using equal probabilities: linear mean swim ($\frac{1}{4}$), circular swim ($\frac{1}{4}$), hyperactive swim ($\frac{1}{4}$), and immotile ($\frac{1}{4}$). The size of the frame was $500 \times 500$ px. The framerate was set at 15 FPS and total of 10 seconds of each image were used for tracking. Twenty (20) different scenarios were generated for each number of cells for a total of 100 images. The background intensity level was 204 (80% of 255) and no noise was added to the video images. The simulation parameters used to generate the images are given in Table 6. Ground truth tracks for each image were provided by the simulation, and were compared to the estimated tracks calculated by NN, GNN, PDAF, and JPDAF algorithms.

**Table 6:** Simulation parameters used for tracking assessment.

Parameter	Value
$r_c$	80 pixel
$f_c$	50 deg/s
$f_s$	4 Hz
*a*	3 pixel
$f_l$	3 Hz
$r_h$	12 pixel
$r_v$	8 pixel
$A_{har}$	0.1
*V*	50 pixel/s
$\sigma _{x_b}, \sigma _{y_b}$	10 pixel

**Table 7:** Tracking performance of NN, GNN, PDAF, and JPDAF algorithms on varying number of cells.

# of cells	MOTP	${\overline{FP}}$	${\overline{M}}$	${\overline{MME}}$	MOTA
**NN**					
20	1.4 px	0.2101	0.0941	0.0003	0.6955
40	1.4 px	0.2935	0.1143	0.0012	0.5910
100	1.4 px	0.3593	0.1609	0.0030	0.4768
200	1.4 px	0.4140	0.2163	0.0061	0.3635
**GNN**					
20	1.4 px	0.0690	0.0812	0.0012	0.8487
40	1.4 px	0.0920	0.0886	0.0028	0.8165
100	1.4 px	0.1317	0.1090	0.0070	0.7524
200	1.5 px	0.1810	0.1343	0.0144	0.6703
**PDAF**					
20	1.4 px	0.2076	0.0994	0.0003	0.6927
40	1.4 px	0.2934	0.1292	0.0009	0.5765
100	1.4 px	0.3636	0.1956	0.0027	0.4381
200	1.5 px	0.4448	0.2748	0.0049	0.2754
**JPDAF**					
20	1.4 px	0.0714	0.0817	0.0012	0.8457
40	1.4 px	0.0969	0.0907	0.0026	0.8098
100	1.4 px	0.1291	0.1099	0.0058	0.7553
200	1.5 px	0.1717	0.1352	0.0118	0.6813

The mean MOTP, ${\overline{FP}}$, ${\overline{M}}$, ${\overline{MME}}$, and MOTA values for 20 different scenarios for each type of images (20, 40, 100, and 200 cells) are shown in Table 7. The values of multi-object tracking precision (MOTP) for the four tracking algorithms were approximately equal regardless of the number of cells in the image. As the number of cells increased, the value of multi-object tracking accuracy (MOTA) decreased and the false positive rate (${\overline{FP}}$), miss detection rate (${\overline{M}}$), and mismatch rate (${\overline{MME}}$) increased.

For large number of cells, GNN and JPDAF algorithms performed better than NN and PDAF tracking algorithms in terms of MOTA. The major factor for this difference was in the false positive rate (${\overline{FP}}$). Comparing NN and PDAF, MOTA for PDAF was lower than NN for all of the testing cases (20, 40, 100, and 200 cells). The GNN and JDPAF tracking algorithms showed similar performance in terms of MOTA. Overall, GNN and JPDAF showed best performance, which was followed by NN. The PDAF tracking algorithm had the worst performance.

## Conclusion

We presented a model of 2-D (top-down) view of a sperm cell and of four (4) different swimming modes generated by observing the swimming paths of real human sperm cells. The simulation opens up opportunities for methodical study and comparison of different semen image processing algorithms, including algorithms for segmentation, localization, and tracking. In our examples, we tested five different segmentation and localization algorithms and used the simulation to rank the algorithms by their performance, obtaining ranking with simulation that appear similar to ranking using real images. In addition, we compared the performance of four different tracking algorithms (NN, GNN, PDAF, and JPDAF) on the simulated images using MOT metrics and have ranked them by their performances.

The simulation models and the software presented in this paper serve as a powerful new tool for developing and enhancing CASA systems and algorithms. Using this new tool, stronger and more robust CASA systems can be developed. The use of such systems is an attractive alternative to manual semen collection and assessment. Specifically, clinicians can generate and demonstrate to students and technicians a variety of images that represent different types of observed semen samples; this task can be accomplished without having to collect, process, and record human semen images. The simulated images can also be used to train technicians, by comparing the results of their analyses of the images to the simulations’ ground truth.

## Supplementary Information

Supplementary Information.

## Data Availability

The raw data, dataset, and software supporting the conclusions of this article are available in the github repositories, [https://github.com/JiwonChoi-NJIT/NJIT_sperm_simulator and https://github.com/moshekam/NJIT-Semen-Images-Data-Fusion-Lab].
