# Supplementary material for: An assessment tool for computer-assisted semen analysis (CASA) algorithms
Source: Sci Rep. 2022 Oct 7;12:16830. doi: 10.1038/s41598-022-20943-9 (PMC9546881; doi:10.1038/s41598-022-20943-9)
Supplement: Supplementary file 1 — Supplementary Information. [file 41598_2022_20943_MOESM1_ESM.pdf]

Additional File 1 - Supporting Information

August 21, 2022

1 Feature Extraction from Human Semen Samples

For the examples discussed in the paper, we have extracted key parameters from two real semen samples of 250 by 250 pixels with duration of 200 frames each. The ground truth locations of each sperm cell in each image were labeled one by one manually by a human.

To obtain the image background B_L, the mean of grayscale intensity values of each pixel was calculated for the duration of 200 frames. Afterwards, the mean grayscale image was convolved with a series of three 7×7 median filters (Ch. 3 in [1]). The output of the 3-stage median filters was used as the image background B_L for the subsequent simulations.

To mimic the noise present in each human semen sample, the noise variance σ_N^2 for each sample was calculated. First, the variance of each pixel for 200 frames was calculated. Afterwards, the median value of the variance of all pixels in the sample was selected to be the noise variance σ_N^2 for the simulation. The noise in the images was assumed to be additive Gaussian. After the simulated images were generated, the images were contaminated by additive Gaussian noise with zero mean and variance σ_N^2.

The size of the sperm head was obtained by segmenting the sperm heads in each frame and finding the median length of the major and minor axes of the segmented heads (Ch. 11 in [1]). To find the two radii of the sperm heads along the major (r_M) and minor (r_m) axis, the median length of each one of the major and minor axes was divided by two. The values of σ_{y_G} and σ_{x_G} of the 2-D Gaussian filter (of the form of equation (1)) were equal to the lengths of radii along the major and minor axes ($\sigma_{y_G} = r_M, \sigma_{x_G} = r_m$).

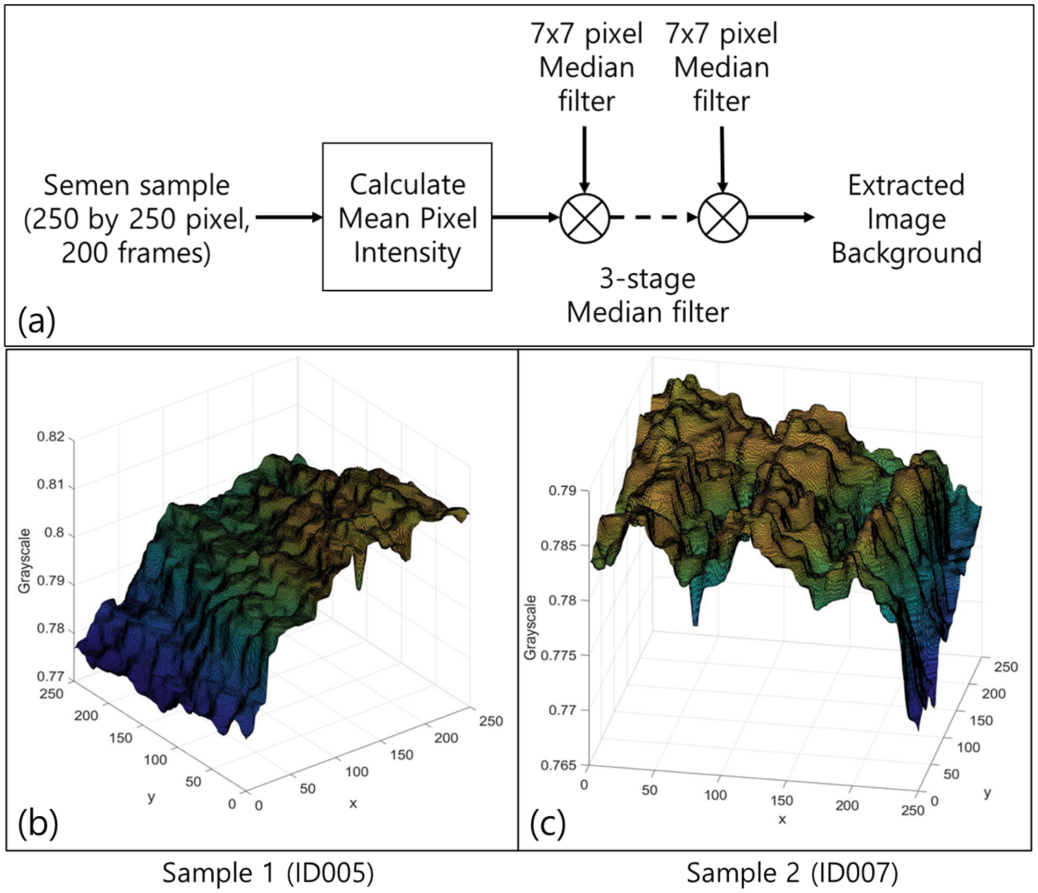

Figure 1: (a) Flowchart of the background extraction process. 3D surface plots of the background model for (b) sample 1 - ID005 and (c) sample 2 - ID007. Grayscale values are normalized to be between 0 and 1.

$$f_1(x,y) = \frac{1}{2\pi\sigma_{x_G}\sigma_{y_G}} exp\left(-\left[\frac{(\frac{x}{\sigma_{x_G}})^2 + (\frac{y}{\sigma_{y_G}})^2}{2}\right]\right). \tag{1}$$

The values of σ_{y_L} and σ_{x_L} of the point spread function f_2 (of the form of equation (2)) were equal to 1.5 times the lengths of radii along major and minor axis ($\sigma_{y_L} = 1.5r_M, \sigma_{x_L} = 1.5r_m$).

$$f_2(x,y) = \max(0, g(x,y)), \tag{2}$$

$$g(x,y) = \nabla^2\left(\frac{1}{2\pi\sigma_{x_L}\sigma_{y_L}} exp\left(-\left[\frac{(\frac{x}{\sigma_{x_L}})^2 + (\frac{y}{\sigma_{y_L}})^2}{2}\right]\right)\right). \tag{3}$$

The value of σ_f for point spread function f_3 was fixed at 1.5.

$$f_3(x,y) = \nabla^2 \left(\frac{1}{2\pi\sigma_f^2} exp\left(- \left[\frac{(\frac{x}{\sigma_f})^2 + (\frac{y}{\sigma_f})^2}{2} \right] \right) \right). \tag{4}$$

In each sample, the number of sperm cells was counted across 200 frames. The number of cells N_C in the simulated image was the maximum number of cells found within 200 frames of each semen sample. The number of non-moving cells N_D was the number of dead/immotile cells found in each sample. The simulation parameters that were extracted from the two samples are shown in table 1. As shown in table 1, sample 1 had three non-moving cells and sample 2 had no non-moving cells.

Table 1: Simulation parameters for segmentation and localization testing

	Noise Variance σ_N^2	Radius r_M (Major axis)	Radius r_m (Minor axis)	Number of cells N_C	Number of non-moving cells N_D
Sample 1	8.22×10^{-6}	2.86 px	1.86 px	10	3
Sample 2	1.03×10^{-4}	2.90 px	1.66 px	8	0

2 Performance Assessment Metrics

The performance of segmentation and localization algorithm on the images was assessed using the optimal subpattern assignment (OSPA) distance [2], and the values of precision and recall. OSPA distance quantifies the cardinality error (differences in number of detection to ground truth) and localization error (differences in distance between the detection and ground truth). The equation of OSPA distance is given as

$$\overline{d}_p^{(c)}(X,Y) := \left(\frac{1}{n} \left(\min_{\pi \in \prod_n} \sum_{i=1}^{m} d^{(c)}(x_i, y_{\pi(i)})^p + c^p(n-m) \right) \right)^{(1/p)}, n \geq m. \tag{5}$$

Here, X is the set of true object locations ($X = \{x_1, ..., x_m\}$). Y is the set of estimated object locations ($Y = \{y_1, ..., y_n\}$). The parameter p is the order. The parameter c is the cut-off distance. m is the number of true objects. n is the number of detected objects. $d^{(c)}(x,y) := \min(c, d(x,y))$ is the distance between x and y with cut-off $c > 0$. For $n < m$, $\overline{d}_p^{(c)}(X,Y) := \overline{d}_p^{(c)}(Y,X)$. We use OPSA parameters $p = 2$ and $c = 20$ pixels. A perfect segmentation and localization algorithm would have

OSPA distance of 0 (no difference between the ground truth and the detections). A poor algorithm would have distance value close to the cut-off distance $c = 20$ (large differences between the ground truth and the detections).

Precision is defined as $TP/(TP + FP)$ and recall is defined as $TP/(TP + FN)$, where TP, FP, and FN refer to true positive, false positive, and false negative, respectively [3]. False positive is defined to be the number of false alarms, and false negative is defined to be the number of missed detections. In the study, true positive is defined to be the number of matches in detection and ground truth within the distance $c = 20$ pixels (cutoff distance). Ideally, the values of precision and recall are 1.

The performance of the tracking algorithm was assessed using the multiple object tracking precision (MOTP) and multiple object tracking accuracy (MOTA) metrics proposed by Bernardin [4]. MOTP is the total error in estimated position of the matched pairs (distance d) over all frames. The unit of MOTP is pixels (px). MOTP is 0 if detected and ground truth tracks are the same. Large MOTP suggests that there are major differences in distance between the detected and ground truth tracks. The cutoff distance c_T, which is defined to be the maximum distance between the matched tracks (ground truth to detected), was 20 pixels. If there were no ground truth tracks within the cutoff distance away from the detected track, the detection was considered to be a false positive. MOTA is derived from 3 different error ratios: false positive rate $\overline{FP}$, missed detection rate $\overline{M}$, and mismatch rate $\overline{MME}$. MOTA is 1 if detected and ground truth tracks are the same. MOTA close to 1 suggests that there are major differences between the detected and ground truth tracks. Values of $\overline{FP}, \overline{M}$, and $\overline{MME}$ are ideally 0.

MOTP is defined as

$$MOTP = \frac{\sum_{i,k} d_k^i}{\sum_k c_k}, \tag{6}$$

where d_k^i the distance between the object o_i and its corresponding hypothesis. k is the frame index and c_k is the number of matched pairs in frame k. If the distance between the estimated track and the ground truth track was beyond the cutoff distance, it was not considered as a matching track. When there were multiple estimated tracks at a given time for a single ground truth track, the Hungarian algorithm was performed to find the most optimal pair based on distance between

the estimate and the ground truth positions [5].

MOTA is defined as

$$MOTA = 1 - \frac{\sum_k FP_k + M_k + MME_k}{\sum_k g_k}, \tag{7}$$

where M_k, FP_k, and MME_k represents the number of missed sequence, false positives, and mismatches in frame k, respectively. g_k is the number of ground truth objects in frame k. MOTA is derived from 3 different error ratios: false positive rate $\overline{FP}$, missed detection rate $\overline{M}$, and mismatch rate $\overline{MME}$.

False positive rate $\overline{FP}$ is the total number of false alarms over the total number of objects present over all frames. Missed detection rate $\overline{M}$ is the total number of misses over the total number of objects present over all frames. Mismatch rate $\overline{MME}$ is the total number of mismatches (change in match of ground truth track to detected track) over the total number of objects present over all frames

$$\overline{FP} = \frac{\sum_k FP_k}{\sum_k g_k}, \quad \overline{M} = \frac{\sum_k M_k}{\sum_k g_k}, \quad \overline{MME} = \frac{\sum_k MME_k}{\sum_k g_k}. \tag{8}$$

3 Additional Segmentation and Localization Algorithm Assessment

In addition to assessment results for the five algorithms on the real and simulated images of sample 1, the OSPA distances, precision and recall rates for varying levels of noise of real and simulated images for sample 2 is shown in figure 2.

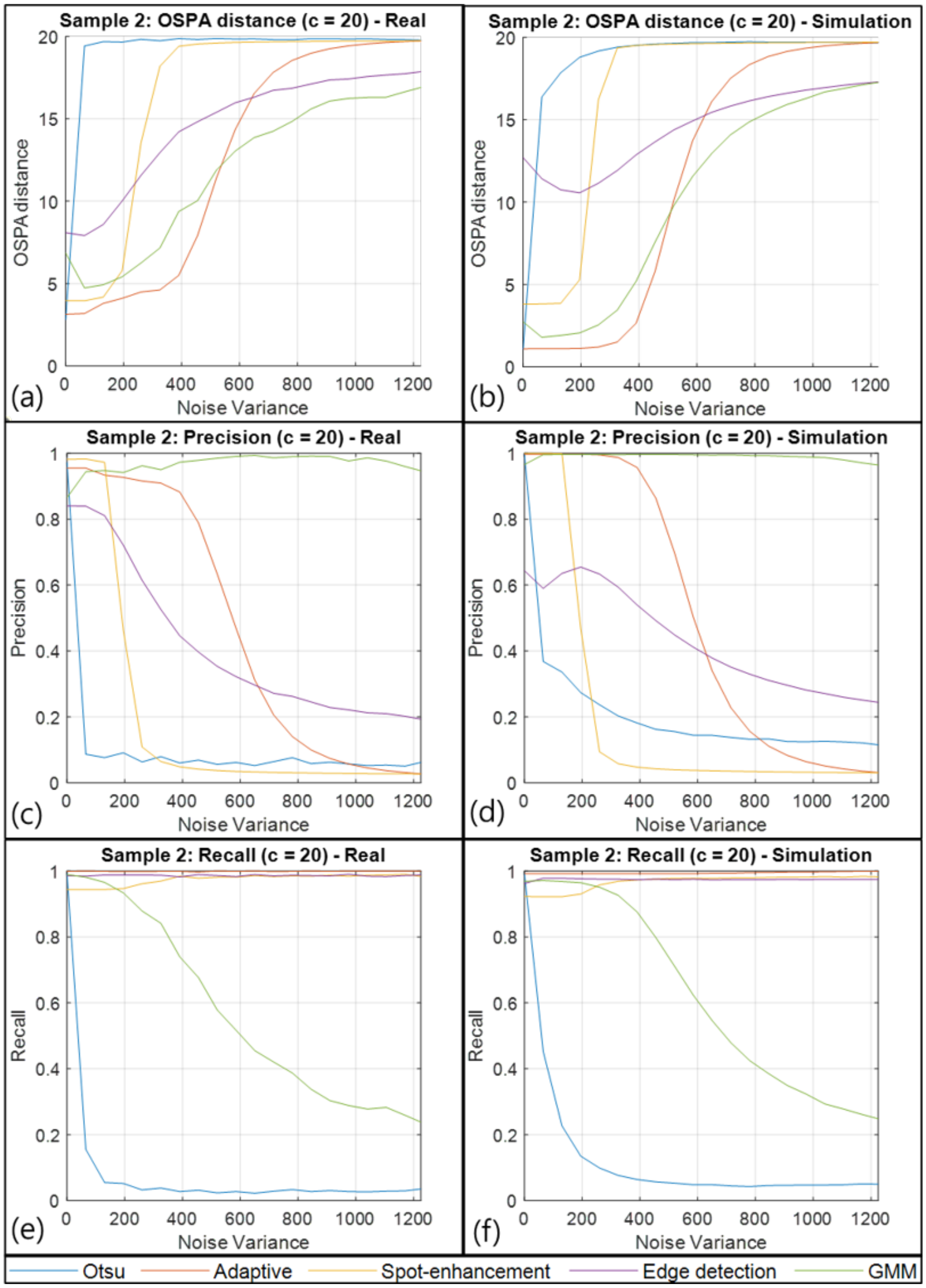

Figure 2: OSPA distance, precision and recall rates of sample 2 for varying levels of additive Gaussian noise ((a,c,e) real, (b,d,f) simulation).

References

[1] Gonzalez, R.C., Woods, R.E.: Digital Image Processing. Pearson, New York (2018)

[2] Schuhmacher, D., Vo, B., Vo, B.: A consistent metric for performance evaluation of multi-object filters. IEEE Transactions on Signal Processing **56**(8), 3447–3457 (2008)

[3] Fawcett, T.: An introduction to roc analysis. Pattern Recognition Letters **27**(8), 861–874 (2006). doi:10.1016/j.patrec.2005.10.010. ROC Analysis in Pattern Recognition

[4] Bernardin, K., Stiefelhagen, R.: Evaluating multiple object tracking performance: The clear mot metrics. EURASIP Journal on Image and Video Processing (2008)

[5] Kuhn, H.W.: The hungarian method for the assignment problem. Naval Research Logistics Quarterly, 83–97 (1955)
